# Molecular differences between cerebral blood volume and vessel size in glioblastoma multiforme

**DOI:** 10.18632/oncotarget.11522

**Published:** 2016-08-23

**Authors:** Dieter Henrik Heiland, Theo Demerath, Elias Kellner, Valerij G. Kiselev, Dietmar Pfeifer, Oliver Schnell, Ori Staszewski, Horst Urbach, Astrid Weyerbrock, Irina Mader

**Affiliations:** ^1^ Department of Neurosurgery, Medical Center University of Freiburg, Freiburg, Germany; ^2^ Department of Neuroradiology, Medical Center University of Freiburg, Freiburg, Germany; ^3^ Medical Physics, Department of Radiology, Medical Center University of Freiburg, Freiburg, Germany; ^4^ Department of Hematology, Oncology and Stem Cell Transplantation, Medical Center University of Freiburg, Freiburg, Germany; ^5^ Department of Neuropathology, Medical Center University of Freiburg, Freiburg, Germany; ^6^ Department of Radiology, Kantonsspital, Medical Center Universtiy of Basel, Switzerland

**Keywords:** radiogenomics, CBV, vessel size imaging, WGCNA, glioblastoma

## Abstract

The purpose of this study was to investigate the molecular background of cerebral blood volume (CBV) and vessel size (VS) of capillaries in glioblastoma multiforme (GBM). Both parameters are derived from extended perfusion MR imaging.

A prospective case study including 21 patients (median age 66 years, 10 females) was performed. Before operation, CBV and VS of contrast enhancing tumor were assessed. Tissue was sampled from the assessed areas under neuronavigation control. After RNA extraction, transcriptional data was analyzed by Weighted Gene Co-Expression Network Analysis (WGCNA) and split into modules based on its network affiliations. Gene Set Enrichment Analysis (GSEA) identified biological functions or pathways of the genetic modules. These were applied on 484 GBM samples of the TCGA database.

Ten modules were highly correlated to CBV and VS. One module was exclusively associated to VS and highly correlated to hypoxia, another one exclusively to CBV showing strong enrichments in the Epithelial Growth Factor (EGF) pathway and Epithelial-to-Mesenchymal-Transition (EMT). Moreover, patients with increased CBV and VS predominantly showed a mesenchymal gene-expression, a finding that could be corroborated by TCGA data.

In conclusion, CBV and VS mirror different genetic pathways and reflect certain molecular subclasses of GBM.

## INTRODUCTION

In recent years, a field of research came to the fore that aims at correlating different genetic imprints of glioblastoma multiforme (GBM) with certain imaging traits in Magnetic Resonance (MR) imaging. Diehn et al. described different “radiogenomic” MR imaging traits corresponding to specific gene expression patterns [1]. For example, contrast enhancement correlated with genes belonging to a hypoxia gene set, containing genes such as *VEGF, Serpine, ADM* or *PLAUR*. Tumors with an increased expression of genes associated with proliferation had a severe mass effect. Two subtypes were identified to have a specific clinical impact: patients with an infiltrative imaging pattern had a worse outcome in comparison to patients with an edematous appearance. Between both groups, typically mesenchymal and proneural genes (OLIG1, OLIG2, SOX6) were differently expressed. Furthermore, an enrichment of gene sets of CNS development and developmental functions were described in the infiltrative subtype. These findings suggest a higher fraction of stem cells in the infiltrative subgroup.

Jamshidi et al. 2010 used the same radiogenomic features for an extended molecular analysis, and added expression datasets and copy number variants to find specific pathway correlations [2]. LTBP1 and RUNX3 were identified in the contrast-enhancing subtype and CHI3L1 was significantly higher expressed in a subgroup associated with a subventricular zone involvement. Two recently published studies showed a connection between hyper-perfusion of GBM and EGFR expression or *EGFRvIII* mutation [3, 4]. By using MR perfusion weighted imaging, an increased cerebral blood volume (CBV) was reported in tumors with EGFR amplification, PTEN deletion, and normal unmethylated O-6-methylguanine-DNA methyltransferase (*MGMT*) [5]. Jain et al. showed an impaired overall survival in GBM with increased CBV. The utilization of the molecular sub-classification of Verhaak [6] improved this connection, but a difference of CBV between the molecular subclasses could not be found [7]. The CBV is calculated from dynamic susceptibility contrast imaging (DSC). An extension of this method allows calculating the size of the capillaries within a range of 10 – 150 μm [8]. Kellner et al. described a strong correlation between histological vessel size in biopsy specimen and vessel size perfusion imaging [9]. Kickingereder et al. identified a decrease of CBV in IDH-mutated patients [10]. In a set of 73 patients with low grade and anaplastic gliomas, the IDH-mutations status could be correctly predicted by CBV in 88%. This study was based only on CBV data and IDH sequencing of 73 patients, whereas an expression analysis without CBV estimation had been performed on 288 patients from the cancer genome atlas (TCGA) [10]. A study by Barajas et al. revealed a different genetic clustering of contrast and non contrast-enhancing tumor by CBV-guided biopsies in a set of 13 GBM [11]. So many genetic observations there are about CBV, so few can be found on the vascular marker “vessel size” (VS). Thus, the purpose of this study was to identify specific genetic profiles and corresponding pathway activation or deactivation associated with the perfusion parameters VS and CBV by an integrative analysis of genetic and perfusion data. The identified genes were to be applied on 484 TCGA data sets for validation.

## RESULTS

**Figure 1:**
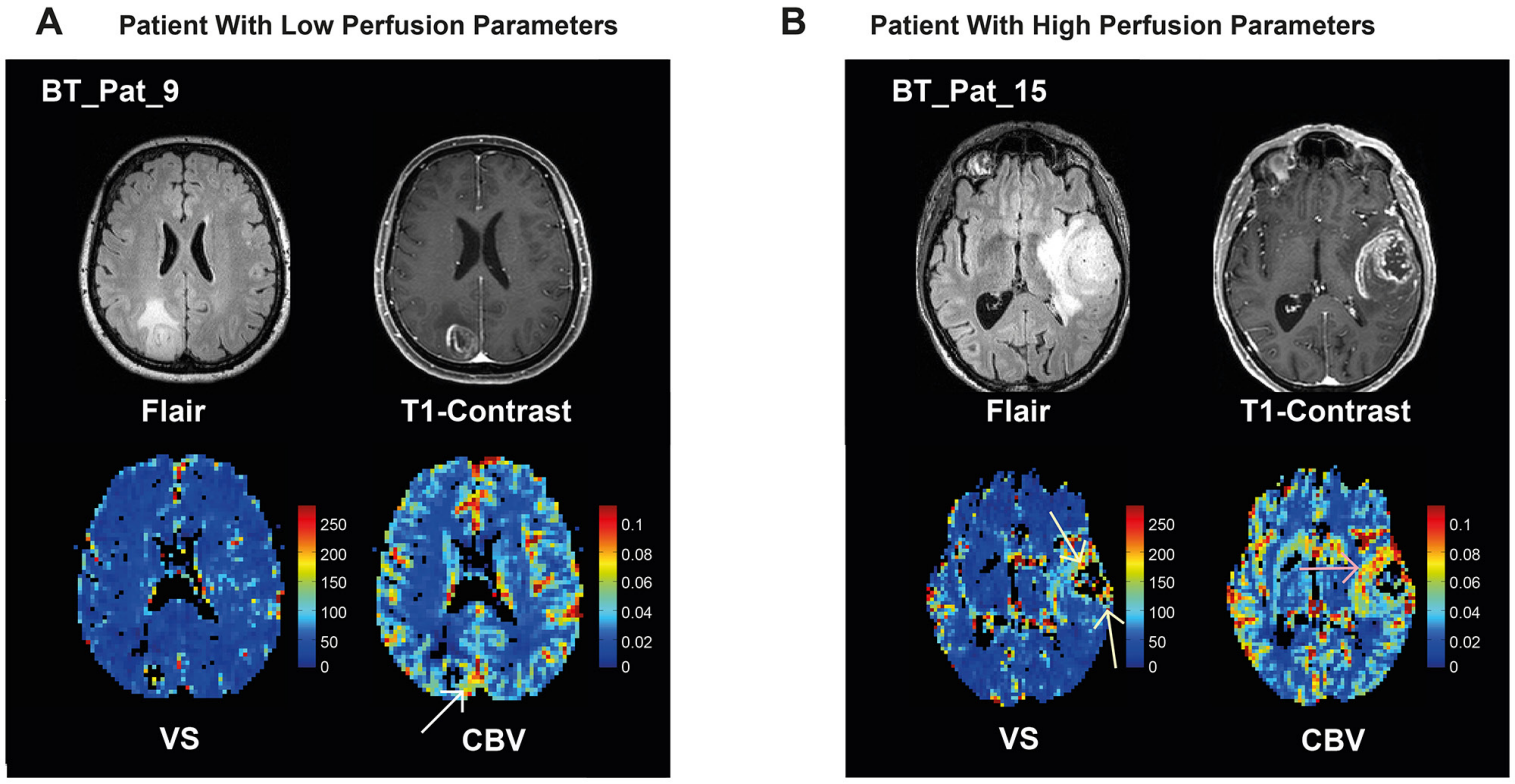
Examples of MR imaging **A**. 63 year-old female patient with a GBM in the right parietal lobe. A FLAIR hyperintense tumor with perifocal edema (top left) with rim-like contrast enhancement and a small central necrosis (top right) is present. The map of vessel size, VS, (bottom left) does not show any increased vessel size. On the CBV map (bottom right) only a shadowy increase of the CBV can be depicted at the rim of the tumor (whitw arrow). **B**. 76 year-old male patient with a GBM in the left temporal lobe. There is a FLAIR hyperintense tumor with perifocal edema (top left) with a thick area of contrast enhancement and a central necrosis (top right). The map of VS (bottom left) reveals increased vessel sizes on the anterior and posterior edges of the tumor (yellow arrows). On the CBV map (bottom right) a strong increase of CBV can be depicted predominantly at the anterior and medial rim of the tumor (red arrow). Both examples show that the VS and CBV calculations result in different non-concordant values in different tumor regions supporting the assumption that both parameters reflect different phenomena of tumor vascularization. The color-bars indicate CBV [ml/100ml tissue] or VS [μm].

In Figure 1, MR images and perfusion maps of two patients with low and high perfusion parameters are given. Detailed data on the patients is given in Table 1.

**Table 1:** Summarized data of all included patients

Sample	IDH1-R132H	VS [μm]	CBV [%]	Expression Subgroup	Age	Sex
BT_Pat_1	wt	103	9	Proneural	65	male
BT_Pat_2	wt	46	2	Proneural	47	female
BT_Pat_3	wt	80	6	Proneural	41	female
BT_Pat_4	wt	80	6	Mesenchymal	55	female
BT_Pat_5	wt	77	6	Proneural	84	female
BT_Pat_6	wt	91	11	Mesenchymal	65	female
BT_Pat_7	wt	76	6	Classical	74	female
BT_Pat_8	wt	102	8	Classical	47	female
BT_Pat_9	wt	52	6	Proneural	63	female
BT_Pat_10	wt	83	5.5	Neural	75	male
BT_Pat_11	wt	99	10	Mesenchymal	78	male
BT_Pat_12	wt	108	8	Mesenchymal	76	male
BT_Pat_13	mut	43	4	Proneural	66	male
BT_Pat_14	wt	58	4	Proneural	79	male
BT_Pat_15	wt	117	7	Mesenchymal	76	male
BT_Pat_16	mut	90	6	Proneural	66	male
BT_Pat_17	wt	72	7	Proneural	61	female
BT_Pat_18	wt	74	5	Proneural	64	male
BT_Pat_19	wt	110	11	Mesenchymal	69	female
BT_Pat_20	mut	103	9	Proneural	77	male
BT_Pat_21	wt	96	7	Mesenchymal	42	male

### Genetics of the study samples

**Figure 2:**
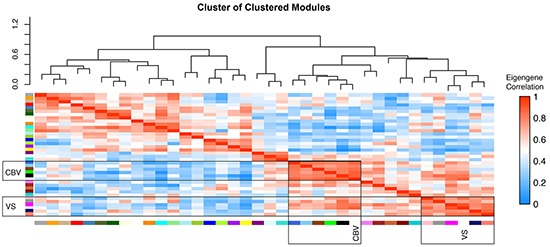
WGCNA cluster of clusters cluster of clusters analysis of modules generated by weighted gene co-expression analysis of genome wide expression analysis. High correlation values are indicated by red, negative correlations by blue colour. CBV and VS associated modules are clustered in differed branches.

Weighted Gene Co-Expression Network Analysis (WGCNA) of the whole array dataset identified 36 modules. A cluster of cluster analysis of these modules is shown in Figure 2. VS and CBV, although thought to be both dependent on tumor vascularization, were surprisingly clustered in different branches. The difference between the molecular backgrounds of both perfusion parameters was subject of further analysis.

**Figure 3:**
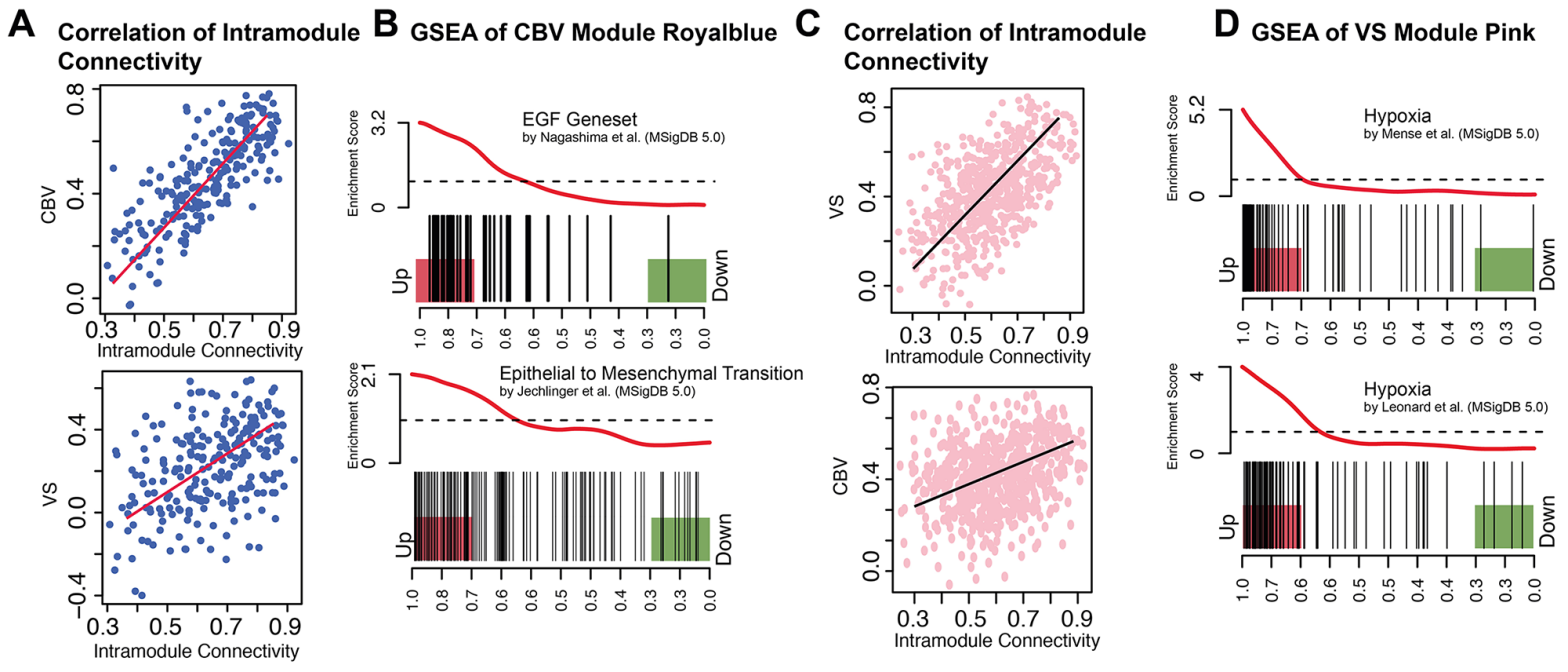
CBV and VS Specific modules **A**. Scatter plot of intramodule connectivity (module royal blue) correlated with CBV (top) and VS (bottom) vectors. **B**. Enrichment plot (GSEA) of module royal blue. EGF-Pathway and Epithelial-to-Mesenchymal Transition are enriched in royal blue hub-genes. **C**. Scatter plot of intramodule connectivity (module pink) correlated with VS (top) and CBV (bottom) vectors. **D**. Enrichment plot (GSEA) of module pink and different hypoxia gene sets. Hub-genes of the pink module are highly enriched in different hypoxia genesets.

Five modules could be identified as being significantly correlated with CBV and VS, respectively, Supplementary Figure S1. Those modules with the most exclusive and highest correlation coefficient for each parameter were taken into further consideration. A module called “royal blue” for CBV had a correlation coefficient of r = 0.78, p = 6E-50, another one called “pink” for VS had a correlation coefficient of r = 0.57, p = 2.3 E-51. To characterize these modules, a pre-ranked permutation based GSEA was performed, Figure 3A-3D.

As shown in Figure 3A, module royal blue was highly correlated to CBV, whereas the correlation with VS was less pronounced (r = 0.53, p = 1.2E-18). The royal blue module showed its strongest association to EGF and EMT pathways in the GSEA (Supplementary Table S1). Gene sets of EGF up-regulation [12] and EMT [13] were enriched with a p_FWER_< 0.01, Figure 3B.

The pink module was strongly correlated to VS (r = 0.57, p = 2.3E-51), but not to CBV (r = 0.25, p = 9.9E-10). The pink module showed its strongest association with hypoxia pathways in the GSEA, Figure 3C. Nine hypoxia gene sets were enriched with a p_FWER_ < 0.01, Figure 3D.

**Figure 4:**
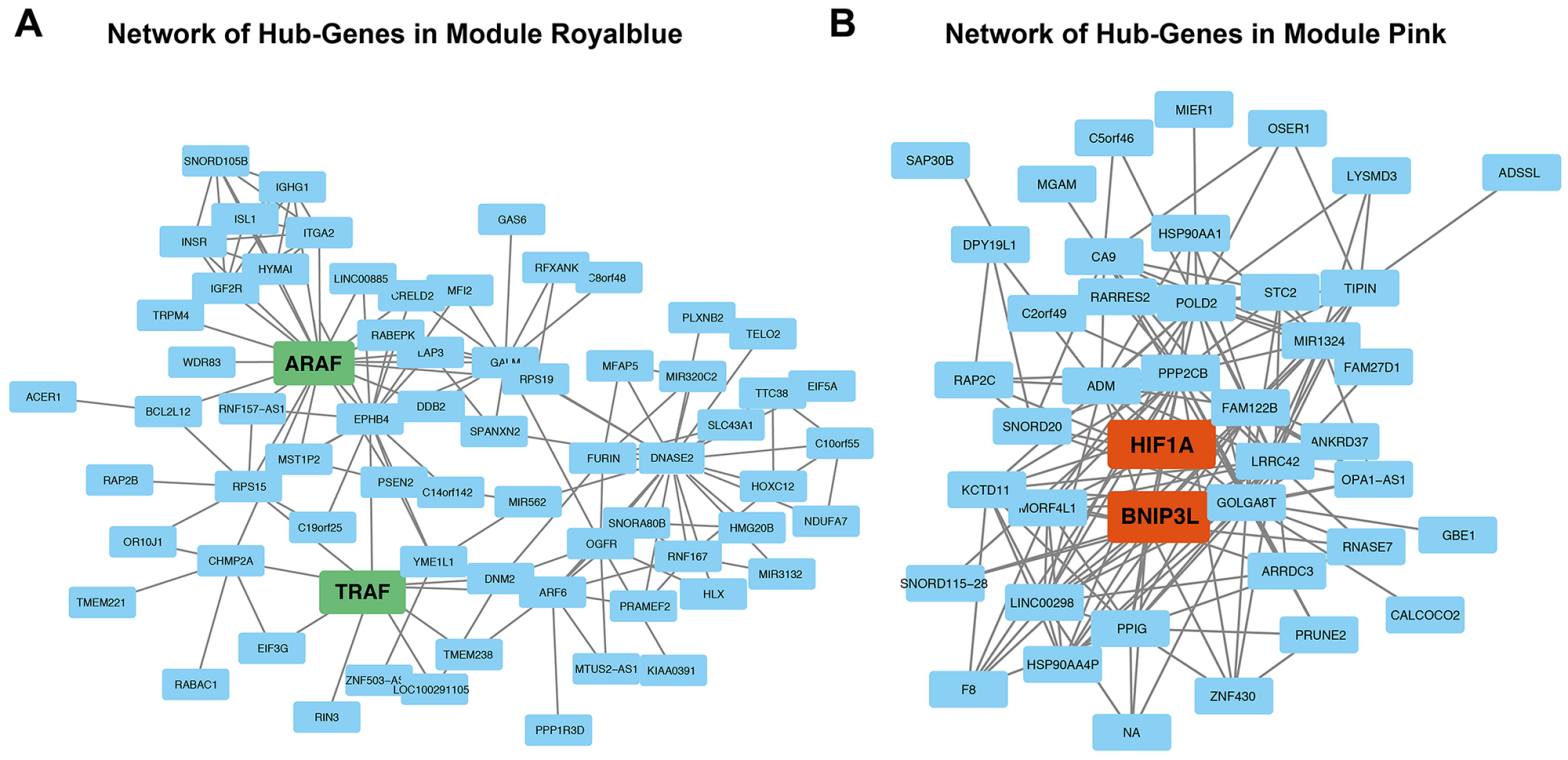
Network analysis **A**. Network analysis of module royal blue. Genes marked in green display module-hub-genes. **B**. Network analysis of module pink. Genes marked in orange display module-hub-genes.

In the royal blue module, *ARAF* and *TRAF* were identified as hub-genes, Figure 4A. *ARAF* takes part of the RAS-MAPK pathway being activated by EGF/EGFR activation and supporting invasiveness of gliomas [14, 15]. The TRAF-family also activate the MAPK-pathway and takes part in angiogenesis [16]. For the pink module, *HIF1A* and *BNIP3L* could be identified as hub-genes, Figure 4B. *HIF1A* is a well-known regulator of hypoxia related pathways [17]. *BNIP3L* has been described as a regulator of hypoxia in conditions of DNA damage [18]. This transcription factor is regulated by methylation and interacts with the MEPK/ERK pathway [19].

**Figure 5:**
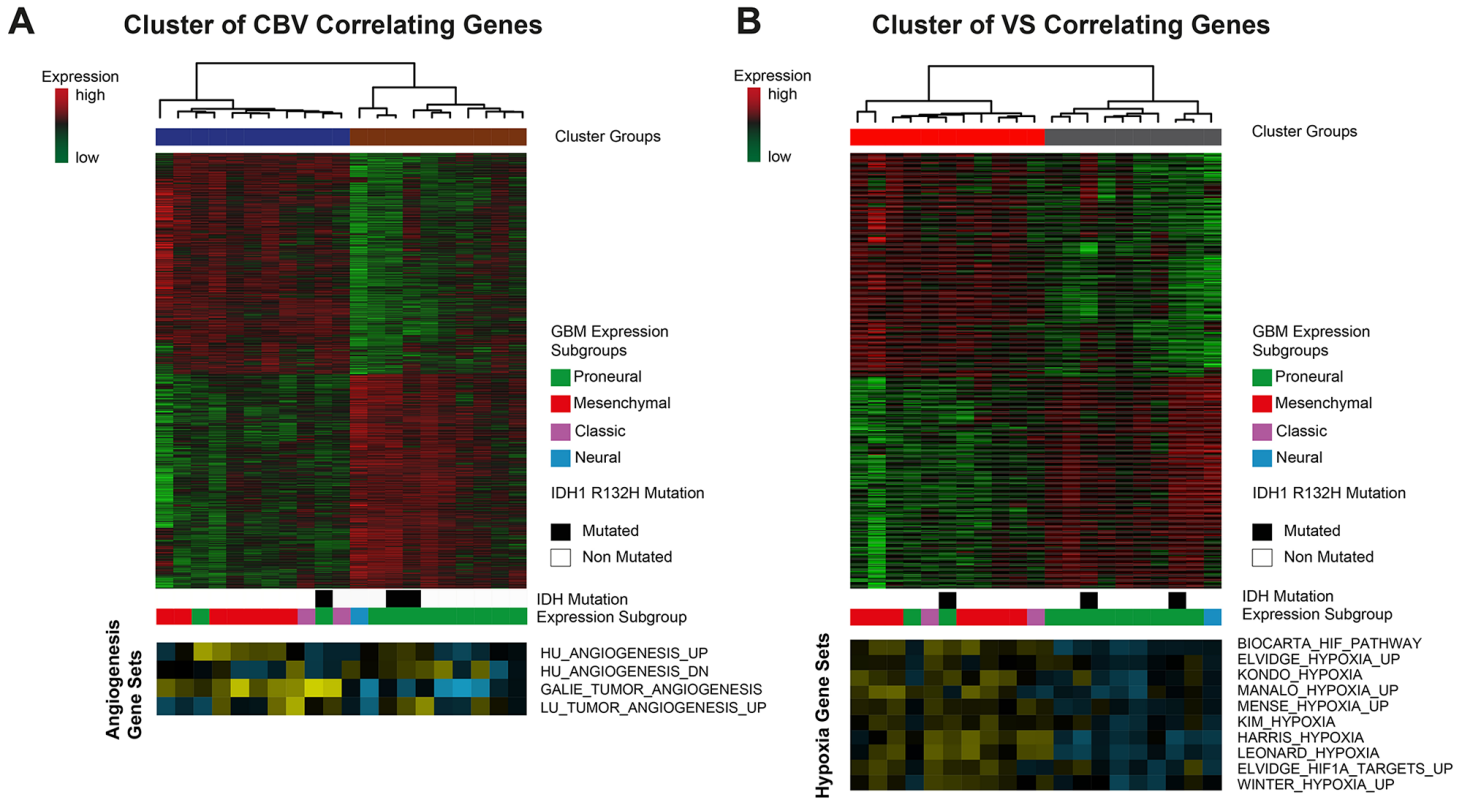
Cluster analysis of CBV and VS correlated genes **A**. CBV-associated genes are clustered by Spearman's rank correlation into two clusters. Bars below the heatmap describe IDH1-status and expression subgroup of each patient. The heatmap at the bottom shows different angiogenesis pathways and their enrichment in each patient. (yellow: high enrichment, blue: low enrichment) **B**. VS-associated genes are clustered by Spearman's rank correlation into two clusters. Bars below the heatmap describe IDH1-status and expression subgroup of each patient. The heatmap at the bottom shows different hypoxia pathways and their enrichment in each patient. (yellow: high enrichment, blue: low enrichment).

Unsupervised clustering of the CBV correlated genes revealed a separation into two clusters (heatmap in the upper row, Figure 5A). In the first (blue) cluster, mean CBV was 8.7 ± 1.4 [ml/100ml tissue], in the second (brown) cluster CBV was 5.1 ± 1.2 [ml/100ml tissue], p_corr_<0.01. In the cluster with high CBV, predominantly patients of the mesenchymal GBM subgroup were present, whereas in the cluster with low CBV, except for one case, only patients of the proneural GBM subgroup could be identified. Additionally, a Wilcoxon model confirmed a significant connection of high CBV values and the mesenchymal subgroup (p<0.05). The heatmap in the lower row displays the enrichment of angiogenesis pathway activation in each patient (Single Sample Gene Set Enrichment Analysis, ssGSEA). Angiogenesis genes were strongly enriched in the high CBV cluster.

Applying the same procedure for the VS correlated genes also resulted in two clusters (heatmap of the upper row, Figure 5B). In the first (red) cluster mean VS was 101.9 ± 7.98 [μm] in the second (gray) cluster VS was 67.36 ± 9.33 [μm], p_corr_<0.01. The cluster with high VS contained predominantly patients of the mesenchymal GBM subgroup, the one with low VS those of the proneural GBM subgroup. This effect was statistically significant (p<0.05). Three patients with IDH 1 mutation were distributed over all clusters. ssGSEA scores of hypoxia pathway enrichment is shown in the bottom row. Patients belonging to the high VS cluster exhibited a strong enrichment of hypoxia pathway genes.

### Comparison with the TCGA validation group

**Figure 6:**
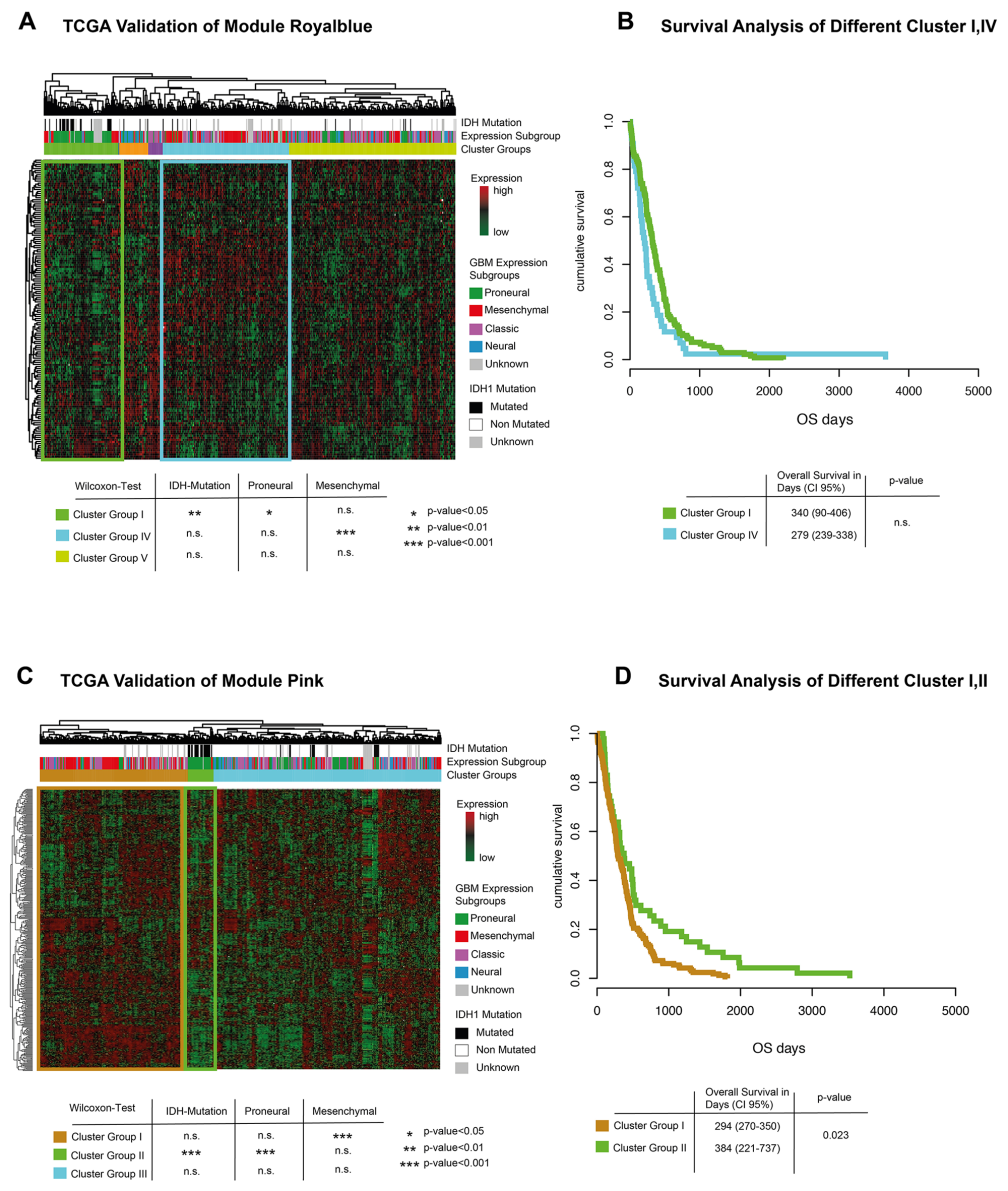
TCGA validation **A**. Genes of module royal blue are extracted and clustered in an unsupervised way. Bars above the heatmap describe IDH1-mutation status and expression subgroup of each patient. Significance values for each cluster and associated genetic subgroups are given in a table below. **B**. Kaplan-Meier plot of cluster-group I and IV. No significant difference could be detected. **C**. Genes of module pink are extracted and clustered in an unsupervised way. Bars above the heatmap describe IDH1-mutation status and expression subgroup of each patient. Significance values for each cluster and associated genetic subgroups are given in a table below. Interestingly, patients with IDH mutation are predominantly found in the clusters with low expression levels. **E**. Kaplan-Meier plot of cluster-group I and II. Cluster-group II showed a significantly better survival.

Modules royal blue and pink were validated in the TCGA database to confirm the findings described above. Hierarchical clustering of genes contained in the CBV related module royal blue showed five different cluster-groups. One out of five was significantly associated with the proneural-, another with the mesenchymal-subgroup, Figure 6A. Survival analysis of both groups showed no significant differences, Figure 6B. Hierarchical clustering of genes contained in the VS related module pink showed three different cluster-groups, Figure 6C. Cluster-group I contained patients with mesenchymal tumors, while cluster-group II was associated to a proneural signature and *IDH* mutation. Survival analysis of both cluster-groups showed a significantly superior overall-survival (Cluster I: 294 days CI-95% 270-350, Cluster I: 384 days CI-95% 221-737, p=0.023) for patients with low expression of VS related genes, Figure 6D. In addition, a validation of CBV and VS correlated genes (Figure 5) is given in the supplementary description.

## DISCUSSION

This study aimed for a direct genetic description of specific expression profiles and pathways being related to CBV and VS, which has only been performed by indirect analysis in the literature so far [10]. A further aim was to identify gene modules being exclusively correlated to CBV *or* VS. Both parameters derive from MR perfusion imaging and are thought to reflect the vascularization of brain tumors. An integrative analysis of transcriptome profiling and imaging parameters was used to identify differences. The gene sets found for both parameters were applied on 484 TCGA samples for further validation.

### Cerebral blood volume

CBV was highly correlated to modules showing a significant enrichment of the EGF pathway. In a further network analysis *ARAF/TRAF* were identified as hub-genes of the highest correlating module (royal blue). *ARAF* is a part of the RAS-MAPK pathway. The RAS-MAPK activation in different cancers is a well-known pathway that supports tumor proliferation, migration and invasiveness [20–23]. *TRAF* belongs to the TNF-signal-cascade and affects EGF pathway by *NFkB* [16]. In a recent study, Kickingereder et al, 2015 [10] described a strong association between CBV and IDH mutation. IDH mutated tumors had significant lower CBV values than IDH wild-type tumors. On consecutive TCGA analysis, IDH wild type tumors showed higher enrichment for hypoxia, angiogenesis and vasculogenesis gene sets than IDH mutant tumors. This lead to the hypothesis, that CBV mirrors this genetic connection, even though transcriptome profiling data of patients with estimated CBV were not available. Our results on direct transcriptome profiling data, however, could show that higher CBV values were more associated to EGF signaling, a RAF/RAS pathway activation and epithelial to mesenchymal transition genes than to hypoxia.

### Vessel-size

VS correlated genes showed a significant enrichment of hypoxia related genes. *HIF1A* and *BNIP3L* were identified as hub-genes in the highest correlating module (pink). *HIF1A*, the hypoxia-inducing factor, is a transcription factor up-regulated under hypoxic conditions. *HIF1A* binds to hypoxia responsive elements (HRE) and activates several genes like *VGFR*. This activation leads to abnormal vascular proliferation in gliomas [17, 24, 25]. So the vessel size (VS) estimated by MR perfusion imaging seems to represent the microvascular environment and abnormal vascular proliferation induced by *HIF1A* activity. The other identified gene was *BNIP3L*, known to be up-regulated in conditions of hypoxia and simultaneous DNA damage [18, 26]. A hallmark of high-grade gliomas is the presence of genetic alterations, including gene mutations and DNA damage [27]. So, these results of the network analysis are in line with typical glioma associated pathways. Unsupervised clustering confirmed the association found between VS and hypoxia related genes.

### Comparison with TCGA data

The replication of the CBV and VS derived clusters on the TCGA database corroborates our results. As shown on our own cohort, an assignment into mesenchymal and proneural signature was possible according to both measures of vascularization. These results are in-line with the findings of Jain et al., who could show a trend to a higher CBV in the classical and mesenchymal subclass than in the neural and proneural subclass [7, 28].

The IDH-mutation, another genetic factor, was predominantly present in one of three patient clusters of VS-correlated genes, which is to say hypoxia-associated genes. This is a finding that was also postulated by Kickingereder et al. [10]. The missing replication of this finding in our own cohort is most probably due to the low number of cases (only three IDH mutant tumors).

### Limitations

The main limitation of this study is the small number of cases. This is the reason, why TCGA data was taken for further validation. Moreover, conservative statistical methods with corrections for multiple testing at each level of analysis were applied. Only family wise error corrected values are reported for the sake of robustness. The wide field of genetic and radiophenomic heterogeneity within GBM tumors was not addressed in this study, as only single tumor biopsies were taken in specific regions based on perfusion imaging. Therefore, findings of this study were to be construed as local radiogenomic results.

In conclusion, this study realized a radiogenomic mapping of glioblastoma multiforme by perfusion imaging parameters (CBV and VS) and genome-wide expression profiling. CBV is a better method to show angiogenesis and EGF pathway activation, whereas VS is more sensitive to detect hypoxia in GBM.

## MATERIALS AND METHODS

### Patients

Twenty-one patients (median age 66 years, range 41 – 84 years, 9 females) with primary glioblastoma multiforme were prospectively included into this study. They underwent surgery at the Department of Neurosurgery between 2012 and 2014. The local ethics committee approved data evaluation, imaging procedures and experimental design (protocol 100020/09 and 5565/15). The methods were carried out in accordance with the approved guidelines. Written informed consent was obtained from all patients.

Inclusion criteria were: (1) age older than 18 years, (2) preoperative MRI with perfusion imaging, (3) intraoperative MRI-guided sampling of tumor tissue from contrast-enhancing tumor, (4) histopathological confirmation of a glioblastoma multiforme (WHO criteria).

### Validation dataset of TCGA data

Publicly available Level 3 TCGA (https://tcga-data.nci.nih.gov/tcga/) data was used for analysis. Data was downloaded at the UCSC Cancer Genome Browser. Only patients with full datasets were included. Expression analyses were based on Agilent array data (TCGA GBM G4502A) for high-grade glioma. Expression data was normalized and log2 transformed. Clustering and further analysis were performed in R-software designed pipeline as described in the WGCNA section.

### Tissue collection and histology

Tumor tissue was sampled from contrast enhancing regions identified by intraoperative neuronavigation (Cranial Map Neuronavigation Cart 2, Stryker, Freiburg, Germany) during resection. The tissue was snap-frozen in liquid nitrogen immediately and processed for further genetic analysis. Tissue samples were fixed using 4% phosphate buffered formaldehyde and paraffin-embedded with standard procedures. H&E staining was performed on 4 μm paraffin sections using standard protocols. Immunohistochemistry was applied using an autostainer (Dako) after heat-induced epitope retrieval in citrate buffer. IDH1 mutation was assessed by immunohistochemistry using an anti-IDH1-R123H antibody (1:20, Dianova).

### MR-imaging

MR imaging was performed on a 3T system (Magnetom TIM TRIO, Siemens, Erlangen, Germany) using a 12-channel head coil. The imaging protocol consisted of a 3D T2-weighted fluid attenuated sequence (repetition time (TR), 5,000ms; effective echo time (TE_eff_), 388ms; inversion time (TI), 1,800 ms; flip angle, variable; pixel size; 1mm^3^), a 3D T1-weighted magnetization prepared rapid gradient echo sequence (TR, 1390ms; TE, 2.15ms; TI, 800ms; flip angle, 15°; pixel size; 1mm^3^) was acquired before and after perfusion imaging with application of 17 ml 0.5 M Gadobenate Dimeglumin (Multihance ®, Bracco, Konstanz, Germany), followed by a chaser of 60 ml NaCl 0.9% solution for perfusion imaging, flow rate 3ml/s. Perfusion imaging consisted of 2D serial, single shot, double-echo readout echo planar imaging (EPI) sequences (TR, 2,000ms; TE_GE_, 21ms; TE_SE_, 94ms, pixel size 2.5 × 2.5 × 5mm^3^) during bolus passage [9].

### MRI post-processing

*Perfusion* data was processed by T1 leakage correction, estimation of the AIF, and calculation of the vessel size and the cerebral blood volume (CBV) as described by Kellner et al. and by the literature cited in there [11]. CBV was normalized to a whole brain median value of 3.2%, equal to the works of Jain et al. [7, 28].

### Genome-wide expression analysis

RNA was prepared using the RNAeasy kit (Qiagen). An amount of 1.5 μg RNA was obtained for expression arrays analysis. Arrays were performed by human genome 2.0 chip (Affymetrix). Raw data was processed, normalized and controlled by R software and the Affymetrix R-package. Different expression analysis and statistical testing (pairwise t-test) were performed by limma R-package.

### Weighted gene co-expression network analysis (WGCNA) and gene set enrichment analysis (GSEA)

WGCNA uses the topological overlapping measurement to identify corresponding modules. The WGCNA analysis is a robust tool for integrative network analysis and was used in several recent studies [29–31]. For the analysis, a signed network analysis with the power of 14 was used to fulfill all criteria of scaled free topology as described by Peter Langfelder. In addition, the branch-cutting algorithm (PAM) with a deep split of two was applied to the analysis. Each identified module was ordered in a “cluster of clusters”-analysis by unsupervised hierarchical clustering. Modules were characterized by their module eigengenes and intramodule connectivity. The intramodule connectivity was correlated to a VS and CBV vector, each. To characterize the correlating modules, a pre-ranked permutation based GSEA [32] was performed (full GSEA data are available in the Supplementary Table S1). Pre-ranked GSEA were performed with 1000 permutations. P-values were calculated by familywise error rate (FWER) [33] which is a robust method for multiples testing. The Molecular Signatures Database version 5.0 was used including pathways gene sets (C2) (http://www.broadinstitute.org/gsea) as input database for this analysis. GSEA plots were visualized by limma R-package (barcodeplot function).

Networks were exported to Cytoscape 2.0 [34] for further visualization. The WGCNA integrated function (exportNetworkToCytoscape) was used to calculate a weighted network by its individual gene connectivity. This analysis identified specific networks for the pink and royal blue module as presented in Figure 4A and 4B. Hallmark genes of each module were characterized by their intramodule connectivity. These potentially important genes were defined as “hub-genes”. In addition a detailed description of WGCNA is given in Heiland et al., 2016 [35].

### Statistical analysis

For non-parametric testing a Wilcoxon model was performed. The significance level was determined with a p-value< 0.05 and a power of 90%. The Kaplan-Meier method was used to provide median point estimates and time-specific rates. The Hazard-Ratio (HR) was calculated by Cox-Regression tests. Statistical tests were performed in R including affiliated packages.

## SUPPLEMENTARY MATERIALS FIGURES AND TABLES




