# Supplementary material for: Molecular differences between cerebral blood volume and vessel size in glioblastoma multiforme
Source: Oncotarget. 2016 Aug 23;8(7):11083–93. doi: 10.18632/oncotarget.11522 (PMC5355248; doi:10.18632/oncotarget.11522)
Supplement: Supplementary file 1 [file oncotarget-08-11083-s001.pdf]

Molecular differences between cerebral blood volume and vessel size in glioblastoma multiforme

SUPPLEMENTARY DESCRIPTION

TCGA validation of CBV and VS correlated genes

In the CBV derived cluster analysis, similar clusters as in the study population could be identified. A cluster with genes correlating with high CBV was clearly visible and contained predominantly GBM of mesenchymal characterization ($p<0.05$). In the remaining cluster, two sub-clusters with a proneural imprint could be distinguished showing similar up and down regulations of genes as in the study population cluster with low CBV. Interestingly, nearly all IDH mutated samples were contained in these subclusters ($p<0.05$), Supplementary Figure S2A.

The VS derived cluster analysis identified three clusters. Most of the samples were in an unspecific cluster. The cluster of negatively correlated genes contained predominantly samples with proneural signature ($p>0.05$), whereas the one with positively correlated genes was attributed to a mesenchymal gene profile ($p>0.05$), Supplementary Figure S2B.

SUPPLEMENTARY FIGURES AND TABLE

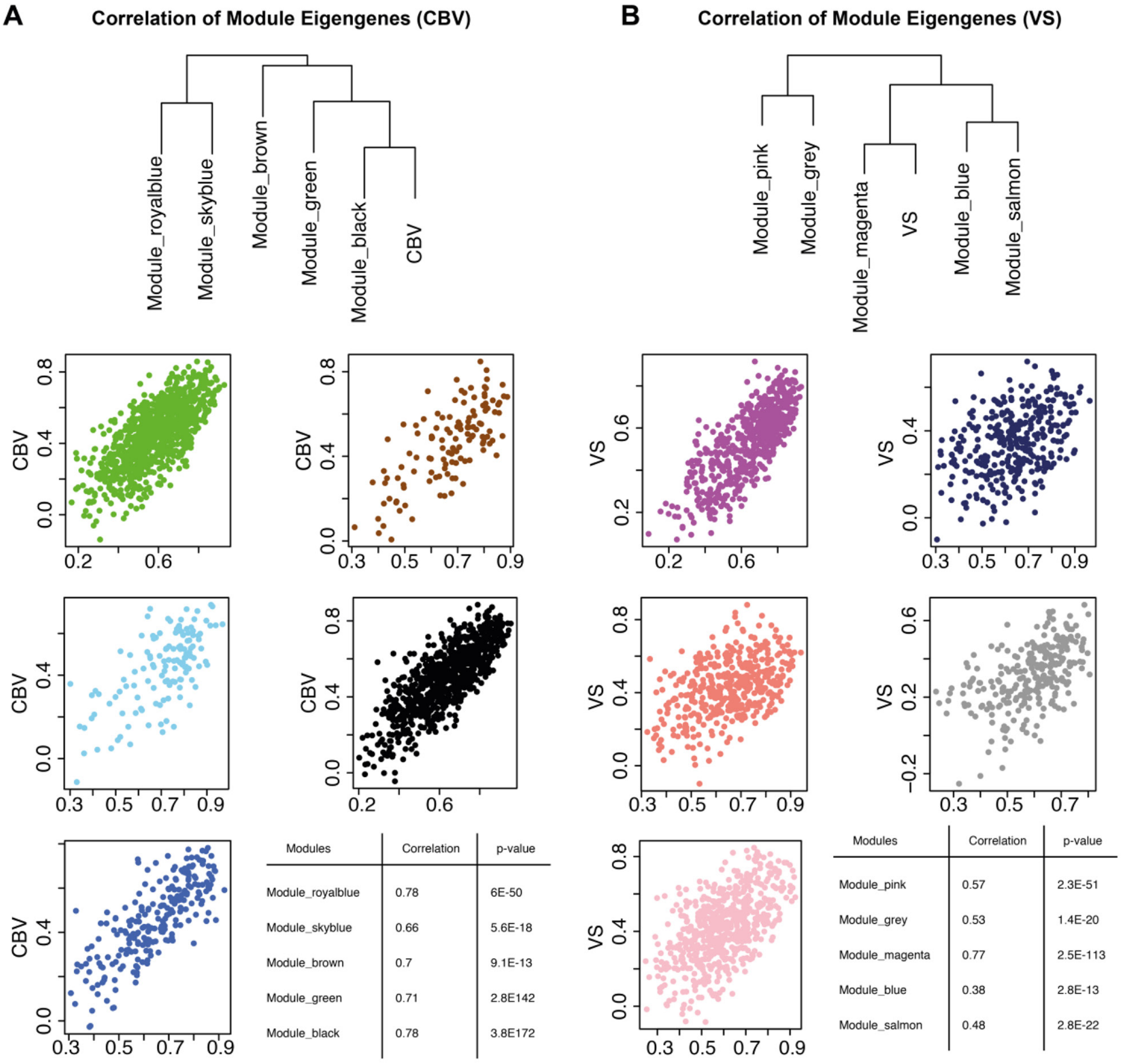

Modules	Correlation	p-value
Module_royalblue	0.78	6E-50
Module_skyblue	0.66	5.6E-18
Module_brown	0.7	9.1E-13
Module_green	0.71	2.8E142
Module_black	0.78	3.8E172

Modules	Correlation	p-value
Module_pink	0.57	2.3E-51
Module_grey	0.53	1.4E-20
Module_magenta	0.77	2.5E-113
Module_blue	0.38	2.8E-13
Module_salmon	0.48	2.8E-22

Supplementary Figure S1: Five modules were significantly correlated with CBV and VS, respectively. Although 10 correlating clusters could be found, only 1 cluster, each, did exclusively correlate with CBV and VS, respectively.

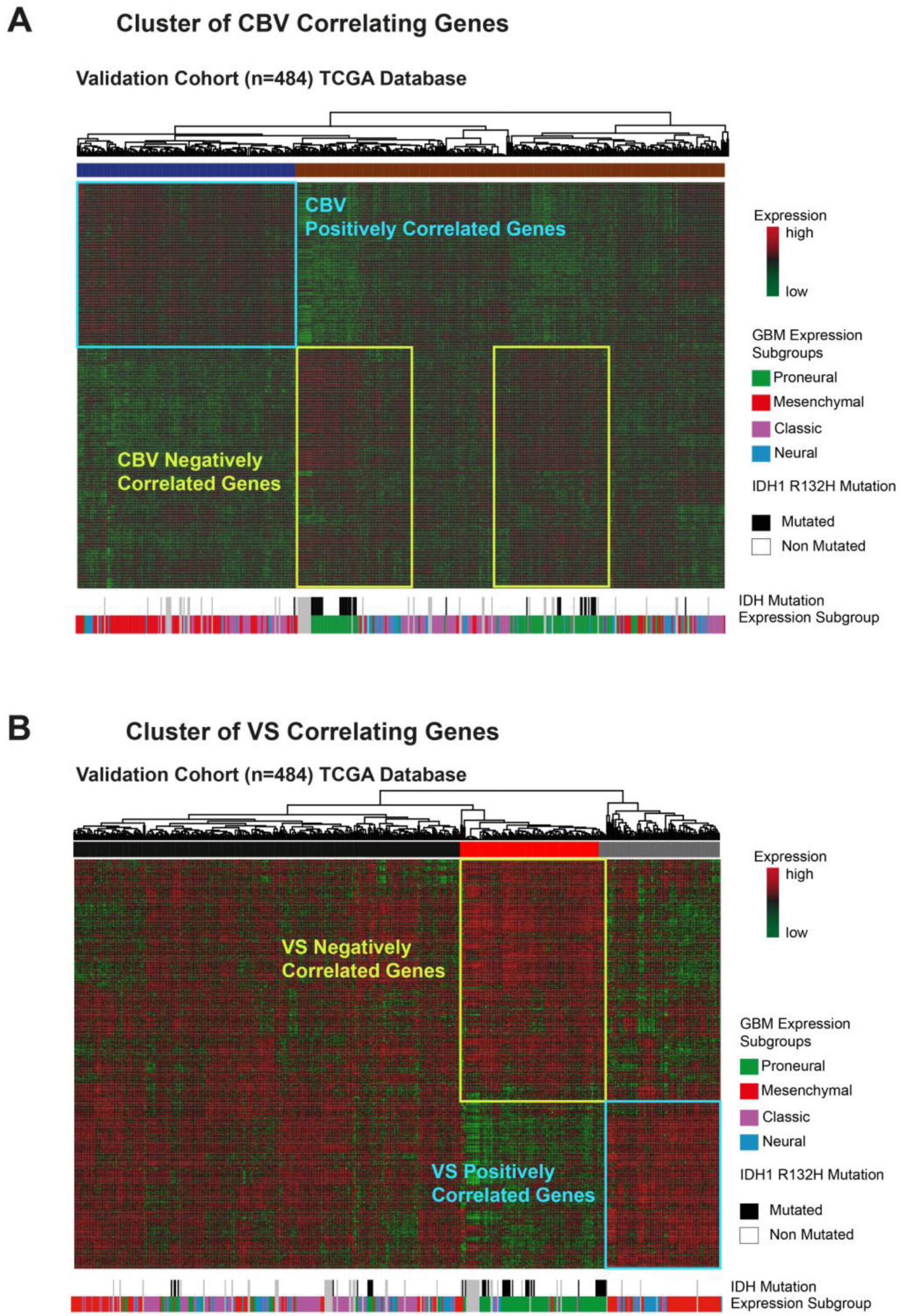

Supplementary Figure S2: A. CBV-associated genes (from Figure 5A) of the TCGA samples were extracted and clustered in an unsupervised way. Bars below the heatmap describe IDH1-mutation status and expression subgroup of each patient. Genes marked in a yellow box were negatively correlated to CBV, those marked in cyan were positively correlated. **B.** VS-associated genes (Figure 5A) of the TCGA samples were extracted and clustered in an unsupervised way. Bars below the heatmap describe IDH1-mutation status and expression subgroup of each patient. Genes marked in a yellow box were negatively correlated to VS, those marked in cyan were positively correlated. Interestingly, patients with IDH mutation are predominantly collected in the clusters with genes being negatively associated CBV and VS.

Supplementary Table S1: Full results of the gene set enrichment analysis.

See Supplementary File 1
